# Supplementary material for: Single-Cell Sequencing of Malignant Ascites Reveals Transcriptomic Remodeling of the Tumor Microenvironment during the Progression of Epithelial Ovarian Cancer
Source: Genes (Basel). 2022 Dec 2;13(12):2276. doi: 10.3390/genes13122276 (PMC9778425; doi:10.3390/genes13122276)
Supplement: Supplementary file 1 [file genes-13-02276-s001.zip › genes-2014008-supplementary.pdf]

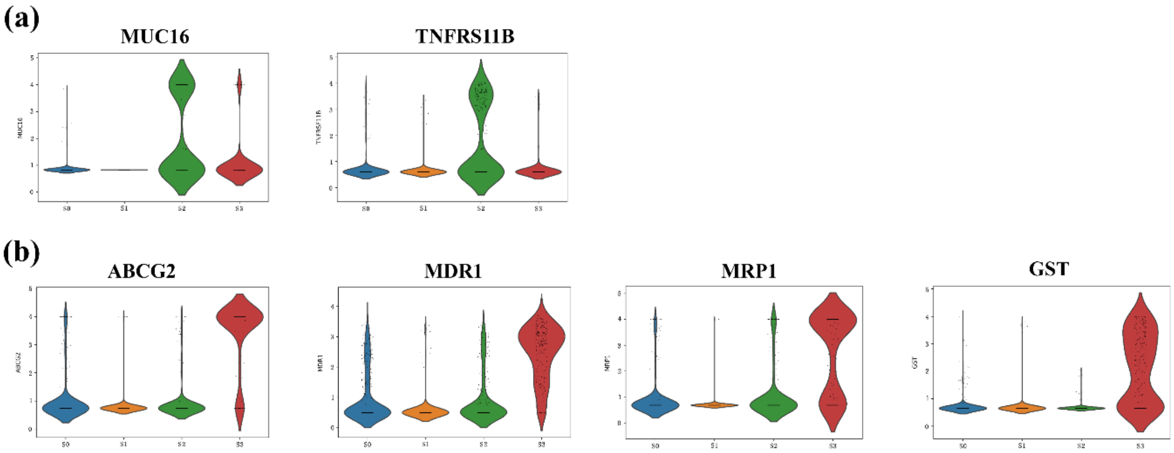

Figure S1. Distinctive subgroups in mesenchymal cancer cells. (a-b) Vlnplots showed the expression of representative genes for immune escape (a) and anti-chemotherapy (b) phenotypes of mesenchymal cancer cells.

Table S1: The information of bulk RNA-seq data used in the research.

Dataset name	Number of samples
GSE14764	80
GSE15622	35
GSE18520	63
GSE19829	28
GSE23554	28
GSE26193	107
GSE26712	195
GSE27651	49
GSE30161	58
GSE3149	116
GSE51373	28
GSE63885	101
GSE65986	55
GSE9891	285
TCGA	565
